# Distinct Motifs in the Intracellular Domain of Human CD30 Differentially Activate Canonical and Alternative Transcription Factor NF-κB Signaling

**DOI:** 10.1371/journal.pone.0045244

**Published:** 2012-09-18

**Authors:** Sarah L. Buchan, Aymen Al-Shamkhani

**Affiliations:** Cancer Sciences Unit, School of Medicine, University of Southampton, Southampton General Hospital, Southampton, United Kingdom; University of Nebraska Medical Center, United States of America

## Abstract

The TNF-receptor superfamily member CD30 is expressed on normal and malignant lymphocytes, including anaplastic large cell lymphoma (ALCL) cells. CD30 transmits multiple effects, including activation of NF-κB signaling, cell proliferation, growth arrest and apoptosis. How CD30 generates these pleiotropic effects is currently unknown. Herein we describe ALCL cells expressing truncated forms of the CD30 intracellular domain that allowed us to identify the key regions responsible for transmitting its biological effects in lymphocytes. The first region (CD30_519–537_) activated both the alternative and canonical NF-κB pathways as detected by p100 and IκBα degradation, IKKβ-dependent transcription of both IκBα and the cyclin-dependent kinase inhibitor p21^WAF1/CIP1^ and induction of cell cycle arrest. In contrast, the second region of CD30 (CD30_538–595_) induced some aspects of canonical NF-κB activation, including transcription of IκBα, but failed to activate the alternative NF-κB pathway or drive p21^WAF1/CIP1^-mediated cell-cycle arrest. Direct comparison of canonical NF-κB activation by the two motifs revealed 4-fold greater p65 nuclear translocation following CD30_519–537_ engagement. These data reveal that independent regions of the CD30 cytoplasmic tail regulate the magnitude and type of NF-κB activation and additionally identify a short motif necessary for CD30-driven growth arrest signals in ALCL cells.

## Introduction

CD30 (TNFRSF8) is a member of the tumour necrosis factor superfamily (TNFRSF), a group defined by the presence of homologous cysteine-rich domains in the extracellular region and which is intimately involved with immune regulation. CD30 exhibits limited expression in health, being predominantly expressed on activated T and B cells. In cancer CD30 is most consistently expressed by Reed-Sternberg cells of Hodgkins lymphoma and a group of neoplasms known as anaplastic large cell lymphoma (ALCL), making it a relatively specific target for immunotherapy [1], [2]. Receptor cross-linking of CD30 activates pleiotropic signals depending on the cell type and agents used. Thus, stimulation of thymocytes, mature T cells, or T-cell hybridomas through CD30 induces apoptosis and cell death [3]–[6] although enhanced proliferation/survival of T cells has also been reported [7]. Similarly, stimulation of ALCL cells via CD30 drives low levels of apoptosis, NF-κB activation, anti-apoptotic signals mediated via the p38 MAPK pathway and growth arrest through the cyclin-dependent kinase inhibitor p21^WAF1/CIP1^ [7]–[12]. In contrast Hodgkins lymphoma cell lines fail to undergo CD30-induced anti-proliferative effects probably due to high constitutive NF-κB activation [8], [9], and instead show a further increase in proliferation in some [7], [11], though not all [8], studies. It is unclear how CD30 is able to activate such a diversity of signals and how this is regulated.

TNFRSF signaling to NF-κB is largely mediated via members of the TNF receptor-associated factor (TRAF) family. Studies in transfected cell lines have shown that TRAFs 1, 2, 3 and 5 interact directly with CD30 via two TRAF-binding sites; residues 561–573 (CD30_561–573_) which bind TRAFs 1, 2, 3 and 5, and residues 578–586 (CD30_578–586_) with specificity for TRAFs 1 and 2 [13]–[16]. In addition, an upstream region of the CD30 cytoplasmic domain may also be involved in transmitting CD30 signals in human embryonic kidney (HEK)293 cell transfectants [16], [17].

Signaling pathways leading to NF-κB activation converge at the level of the IKK complex which facilitates degradation of IκB-family proteins resulting in release and preferential nuclear localisation of the previously IκB-bound NF-κB transcription factor. NF-κB exists as a homo- or hetero-dimer comprised from 5 sub-units (p65 (also called RelA), RelB, cRel, p100/p52 and p105/p50) defined by the presence of the Rel homology domain, which enables DNA binding and interaction with IκB [18]. Canonical NF-κB activation utilises an IKKβ-containing complex to drive phosphorylation and degradation of the classical IκB proteins IκBα, IκBβ and IκBε resulting in predominantly release of the p65/p50 dimer [18]. A subset of the TNFRSF, including CD30, CD40 and BAFFR, additionally activate the alternative NF-κB pathway [19]. For CD40 and BAFFR this is achieved subsequent to TRAF2, cIAP-1 and cIAP-2 dependent degradation of TRAF3 [20]. In resting cells TRAF3 targets the IKK kinase NIK for proteasomal degradation; thus, receptor driven TRAF3 degradation allows NIK stabilisation and subsequent NIK-mediated phosphorylation/activation of homodimeric IKKα [20], [21]. Activated IKKα facilitates degradation of the C-terminal region of p100 (which acts as an IκB protein) to release the p52 fragment and allow nuclear entry of RelB/p52, the prototypical alternative NF-κB isoform. While the canonical NF-κB pathway is associated with proliferative and pro-survival signaling, non-canonical NF-κB activation regulates lymphogenesis during development. However, some evidence additionally points to a role for elements of the non-canonical NF-κB pathway in enhancing canonical NF-κB activation [22]–[24].

Previous reports which sought to identify the cytoplasmic regions that transmit CD30 signaling utilised ectopic expression of constitutively active (ligand-independent) CD30 variants in HEK293 cells, a cell type in which CD30 expression has never been observed [13], [15]–[17]. Consequently, the role of the various cytoplasmic sub-domains of CD30 in mediating its specific effects in haematopoietic cells, including the induction of cell cycle arrest remains unknown. To gain insight into how CD30 induces pleiotropic signals in haematopoietic cells, we generated ALCL cell lines expressing chimeric forms of CD30 incorporating truncated versions of its intracellular domain fused to the extracellular region of the related TNFR superfamily member 4-1BB (TNFRSF9). By studying the effects of 4-1BBL stimulation on these cells, we report that the intracellular domain of CD30 comprises at least two distinct functional regions with independent and disparate signaling activity.

## Materials and Methods

### Cell Lines and Plasmids

ALCL cell lines Karpas-299 (from DSMZ, Germany, and authenticated by multiplex PCR, cytogenetics and mycoplasma testing) and Michel (kindly provided by Prof. W. Murphy, University of Nevada, NV, USA and described in reference [25]) and subsequently authenticated as mycoplasma negative (Endosafe-PTS, Charles River, U.K.) and CD30- and ALK-rearrangement positive (FISH kindly performed by Dr. F. Ross, Wessex Regional Genetics Laboratory, Salisbury, U.K,) were maintained as described [10], [12]. Cells were expanded, aliquoted and frozen and maintained for less than 6 months in culture. For chimeric receptors, RNA was extracted (RNeasy; Qiagen, Crawley, U.K.) and cDNA synthesised (Superscript® III First Strand Kit; Invitrogen, Paisley, U.K.) from Con-A treated murine splenocytes (m4-1BB) and Karpas-299 cells (hCD30). Sequence encoding the extracellular and transmembrane domains of 4-1BB flanked by *Bgl*II and *Xho*I sites was amplified using primers *Bgl*II4-1BBF; TTT*AGATCT*CATTTCGCCATGGGAAACAAC and 4-1BBtailless*Xho*IR; TTT*CTCGAG*TTAGATCCATTTGAGCACAGA, cloned (Zero-Blunt TOPO PCR Cloning Kit; Invitrogen) and ligated into pMSCV2.2 to generate pTL. For pFL, overlapping sequences were spliced encoding firstly, the extracellular and transmembrane regions of m4-1BB and a region of the hCD30 cytoplasmic tail and secondly, a region of m4-1BB and the entire hCD30 cytoplasmic region, amplified by PCR using forward (*Bgl*II4-1BBF and CTGTTCTCTGTGCTCCACCGGAGGGCCTGC) and reverse (GCAGGCCCTCCGGTGGAGCACAGAGAACAG and CD30*Xho*IR; TTT*CTCGAG*TCACTTTCCAGAGGCAGC) primers, and m4-1BB and hCD30 templates, respectively. Plasmids p519, pD1 and pD1D2 were constructed using pFL as template, *Bgl*II4-1BBF as forward primer and TTT*CTCGAG*TCAGATGTAGATTTTCTC (p519); TTT*CTCGAG*TCACCGGCCCTCCGGCAG (pD1) and TTT*CTCGAG*TCAGACATCGCTGCAGCT (pD1D2) as reverse primers. For pD2D3, sequence encoding m4-1BB extracellular and transmembrane domains, hCD30 cytoplasmic tail to residue 519 and a short sequence from D2 of hCD30 were amplified from pFL using primers *Bgl*II4-1BBF and TGGCCCCGCCAGGCCGATGTAGATTTTCTC and spliced with sequence encoding hCD30 residues 515–519, 538–595 and *Xho*I restriction site, similarly synthesised using primers GAGAAAATCTACATCGGCCTGGCGGGGCCA and CD30*Xho*IR. All products were inserted into pMSCV2.2 upstream of internal ribosomal entry sequence (IRES) and GFP and inserts sequenced.

### Retroviral Transduction and Flow Cytometry

Plasmid DNA and pCL-Ampho (Imgenex, San Diego, CA, U.S.A.) were transfected into amphotropic Phoenix cells (LGC Standards, Middlesex, U.K.) using calcium phosphate (with 25 µM chloroquine) or Effectene Transfection Reagent (Qiagen). Virus was collected at 48 hours, spin-infected onto ALCL cell lines and GFP^+^ cells sorted using a FACsAria. Surface 4-1BB expression was confirmed by flow cytometry (FACSCalibur; Becton Dickinson, Oxford, U.K.) using rat anti-mouse 4-1BB (LOB12.3) or rat anti-mouse OX40 control (OX86; both in house) and allophycocyanin-conjugated anti-rat antibody (Jackson ImmunoResearch Laboratories, Suffolk, U.K.).

### Ligands and Blocking Reagents

Soluble recombinant CD30L and 4-1BBL immunoglobulin fusion proteins have been described [12], [26]. ALCL cells were stimulated with 10 µg/ml CD30L, 4-1BBL, human immunoglobulin or CD70 [27] (controls) or titrated doses of 4-1BBL. For blockade of signaling pathways, 10 µM TPCA-1 (2-[(aminocarbonyl)amino]-5-(4-fluorophenyl)-3-thiophenecarboxamide), UO126, SB203580 or SP600125 (or dimethyl-sulfoxide as a control) were used in the presence of 25 µM pan-caspase inhibitor zVAD-FMK (all Calbiochem, Darmstadt, Germany).

### Cell Cycle and Growth Arrest Analysis

**Figure 1:**
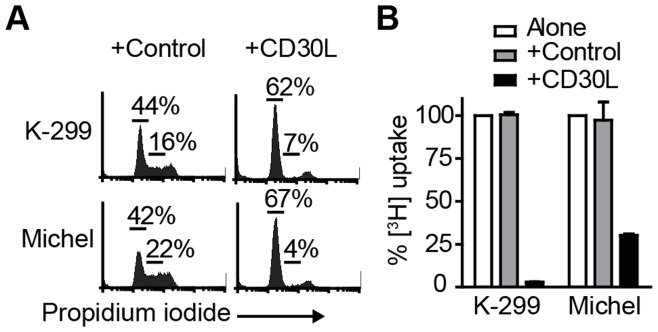
CD30L triggers growth arrest in two ALCL cell lines. Karpas-299 (K-299) or Michel cells were incubated alone, with control (human IgG) or CD30L for (A) 24 hours and DNA content determined by propidium iodide staining; numbers indicate the percentage of cells in G_0_/G_1_ (left) or S-phase (right), or (B) 72 hours with [^3^H]-thymidine added for the final 16 hours. Bar graphs show the mean (+/−SEM) percentage of [^3^H]-thymidine uptake from triplicate wells in control or CD30L-stimulated cells; data are expressed as a percentage of [^3^H]-thymidine-uptake relative to untreated cells (normalised to 100%) and are representative of at least 3 independent experiments.

**Figure 2:**
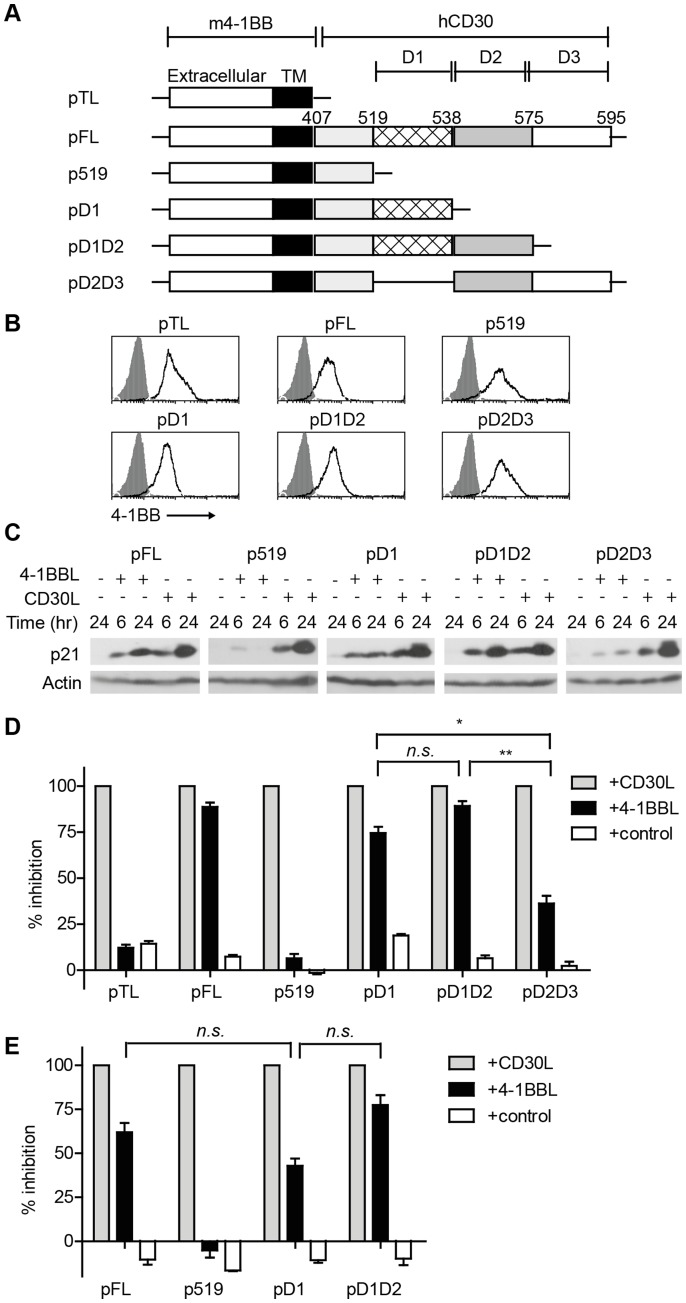
Signals from CD30 D1 drive increased expression of p21^WAF1/CIP1^ and growth arrest in ALCL cells. (A) Schematic representation of all retroviral constructs showing the position of domains (D1, D2 and D3) of hCD30 downstream of m4-1BB extracellular and transmembrane (TM) domains. Numbers indicate CD30 residues relative to their position in full-length hCD30. (B) Karpas-299 cells were stably transduced with the constructs indicated and surface expression of m4-1BB detected by flow cytometry (open histograms); filled histograms represent staining with isotype control. (C) Karpas-299 transductants were stimulated for 6 or 24 hours with 4-1BBL or CD30L or left untreated for 24 hours and p21^WAF1/CIP1^ detected by immunoblot; actin is shown as a loading control. Blots are representative of two independent experiments. To determine the role of CD30 domains in triggering growth arrest, Karpas-299 (D) or Michel (E) cells transduced with the plasmids indicated, were stimulated for 72 hours and [^3^H]-thymidine uptake determined as a measure of cell cycling. Data are normalised to maximum growth inhibition (that induced by CD30L, set at 100%) and to minimum inhibition (medium alone; 0%) to show relative % inhibition induced by CD30L, 4-1BBL or control (CD70). Data in panels D and E are pooled from 3 of 3 (D) or 2 of 2 (E) experiments and show means and SEM. One-way ANOVA was performed on 4-1BBL-stimulated data sets across all experiments and was significant for Karpas-299 (p<0.0001) and Michel (p<0.018) cells. Statistical analyses using the Students two-tailed t-test were therefore performed comparing the means of individual groups; *p<0.05, **p<0.005, n.s. p>0.05.

Cell cycle analysis [12] and [^3^H]-thymidine uptake assays [28] were performed as described. Data are expressed as either mean % [^3^H]-thymidine uptake of triplicates relative to untreated cells (set at 100%; Figure 1), or as % growth inhibition normalised to that induced by CD30L (set at 100%; Figure 2).

### Immunoblot

For immunoblotting, 0.5–1×10^6^ cells were lysed in protein solubilization buffer (PSB) [12], reduced, denatured and resolved on 10% SDS-PAGE gel. Cytoplasmic and nuclear lysates were prepared by differential detergent fractionation using published methods [29]. Proteins were transferred to Immobilon-P membranes (Millipore, Billerica, MA, U.S.A.), incubated with antibodies against IκBα (C-15), p100/p52 (C-5), actin (C-11; all Santa Cruz Biotechnology, Santa Cruz, CA, U.S.A.), p21^WAF1/CIP1^ (DCS60), phospho-IκBα (5A5), p65, p105/p50 or lamin A/C (4C11; all Cell Signaling Technology, Danvers, MA, U.S.A.) and visualised with HRP-conjugated anti-rabbit, anti-mouse (both GE Healthcare, Bucks, U.K.) or anti-goat (Santa Cruz Biotechnology) secondary antibodies using Supersignal West Pico Chemiluminescent Substrate (Thermo Fisher Scientific, Loughborough, U.K.).

### Quantitative PCR

IκBα and p21^waf1^ transcripts from stimulated or control ALCL cells were quantitated using quantitative reverse-transcription-PCR (qRT-PCR) as described [30] using TaqMan gene expression assays Hs00153283m1 (IκBα) and Hs00355782m1 (p21^waf1^) and HPRT (TaqMan assay Hs02800695m1; all Applied Biosystems, CA, U.S.A.) as a reference gene. Fold expression relative to untreated cells was determined using the ΔΔ^CT^ method.

## Results

### CD30L Induces Growth Arrest in ALCL Cells

We and others have previously reported that exposure of the ALCL cell line Karpas-299 to either membrane-expressed or soluble CD30 ligand (CD30L, CD153) induces cell cycle arrest [10], [12]. To investigate whether CD30L induces growth arrest in another ALCL cell line, we incubated the CD30-expressing ALCL cell line, Michel with CD30L. Pronounced growth arrest at the G_0_G_1_ stage was repeatedly observed (Figure 1A) with greater than 70% reduction in cell proliferation in longer-term cultures (Figure 1B).

### Upregulation of the Cell Cycle Inhibitor p21^WAF1/CIP1^ and Growth Arrest is Initiated through CD30_519–537_ in ALCL Cells

CD30-induced growth arrest in Karpas-299 cells correlates with increasing expression of the cyclin dependent kinase inhibitor p21^WAF1/CIP1^ [8], [10], a known target of NF-κB [10], [31]. Three regions of the CD30 cytoplasmic tail independently induce NF-κB activation in HEK293 cells [13]–[17]. To determine if these regions upregulate p21^WAF1/CIP1^ and thereby induce growth arrest in ALCL cells, we constructed a panel of retroviral plasmids encoding chimeric receptors which incorporate the extracellular and transmembrane domains of murine 4-1BB alone or in frame with full-length or truncated segments of the human CD30 cytoplasmic tail to enable m4-1BBL-induced CD30 signaling (Figure 2A). Receptors were designed based on domains 1, 2 and 3 (henceforth D1, D2 and D3) described by Horie *et. al.* (1998) [17]. Domains D2 and D3 each incorporate one of the two previously defined TRAF-binding sites while D1 represents a non-TRAF binding region [14], [16], [17]. In the present study we have reduced D1 from CD30_500–538_ to CD30_519–537_ based on data indicating an absence of NF-κB activity from CD30_500–519_ [17]. Retroviral constructs were stably introduced into Karpas-299 cells and similar surface expression of the chimeric transgenes was confirmed by detection of murine 4-1BB protein (Figure 2B), and mRNA for each of the receptor variants by RT-PCR (data not shown).

Cells expressing a chimeric receptor incorporating the full-length CD30 intracellular tail (pFL; Figure 2C) upregulated p21^WAF1/CIP1^ protein by 6 hours following 4-1BBL stimulation; in contrast little p21^WAF1/CIP1^ was induced by cells expressing the minimal p519 receptor as anticipated. Unexpectedly, p21^WAF1/CIP1^ expression was greater when signaling emanated from D1 compared with D2D3 (Figure 2C; compare centre and far right panels). In addition, the D1D2-containing receptor stimulated slightly increased p21^WAF1/CIP1^ expression compared with D1 (Figure 2C, compare central and fourth panels). Comparable increases in p21^WAF1/CIP1^ protein were detected in all cell lines after incubation with CD30L, confirming that p519 and pD2D3-expressing cells retained competency for endogenous signaling via CD30. Together these data reveal that the majority of p21^WAF1/CIP1^ protein synthesis is initiated by signals from the D1 domain, with a small contribution from D2.

To determine whether differential expression of p21^WAF1/CIP1^ correlates with induction of tumour growth arrest, cell lines were incubated alone, with irrelevant ligand (control), 4-1BBL or CD30L and [^3^H]-thymidine uptake used as a measure of cell proliferation (Figure 2D). As expected, substantial growth inhibition was observed in Karpas-299 cells expressing pFL but not pTL or p519 after 4-1BBL incubation (means and SEM across 3 experiments; 88.7% +/−4.1%, 12.3% +/−2.8% and 6.5% +/−4.1% inhibition respectively). Notably the division of 4-1BBL-stimulated pD1 and pD1D2 expressors was also inhibited (means of 74.6% +/−5.8% and 89.2+/−4.6% inhibition respectively across 3 experiments) in line with the high levels of p21^WAF1/CIP1^ protein induced in these cells (Figure 2C). In contrast, 4-1BBL-stimulated Karpas-299 cells that expressed pD2D3 showed relatively little growth arrest (mean of 36.3% +/−7.2% inhibition across 3 experiments), significantly lower than that induced by signals through either pD1 or pD1D2 again reflecting the low expression of p21^WAF1/CIP1^ protein by activated pD2D3-expressing cells compared with pD1 and pD1D2 expressors (Figure 2C).

To determine if signals from CD30 D1 drive growth arrest in other ALCL cells, Michel cells were similarly transduced with pFL, p519, pD1 or pD1D2 plasmids and comparable expression of surface 4-1BB confirmed by flow cytometry (Figure S1). Again, pFL, pD1D2 and pD1, but not p519, transductants showed 4-1BBL-induced growth inhibition (Figure 2E). Flow cytometric DNA content analysis confirmed that reduced [^3^H]-thymidine uptake after 4-1BBL-stimulation was due to growth inhibition rather than apoptosis (Figure S2 and data not shown). Together these data reveal a novel role for the D1 region (residues 519–537) of CD30 in driving upregulation of p21^WAF1/CIP1^ and retardation of ALCL cell growth.

### Distinct Cytoplasmic Regions of CD30 are Required for Activation of the Alternative and Canonical NF-κB Pathways

**Figure 3:**
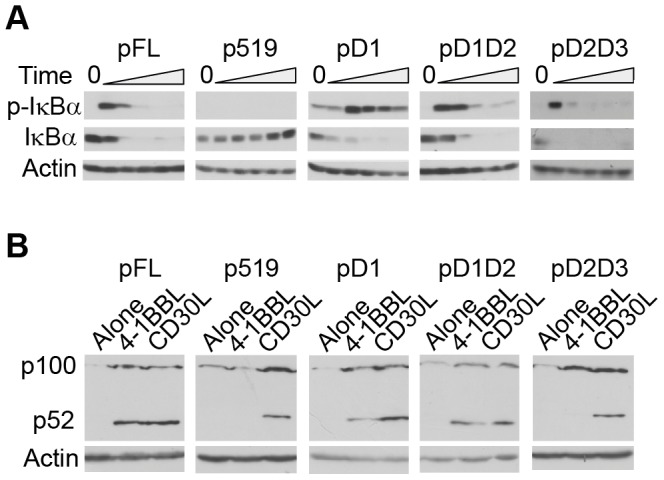
CD30 D1 is necessary and sufficient for activation of the alternative NF-κB pathway. (A) Karpas-299 transductants were incubated alone (0) or with 4-1BBL for 5, 10, 15, 20 or 30 mins and expression of phospho-IκBα (upper panels) or total IκBα (central panels) determined by immunoblot. (B) Karpas-299 transductants were incubated for 24 hours alone or in the presence of 4-1BBL or CD30L; p100 and its degradation product p52 were detected by immunoblot. Blots in (A) and (B) are representative of 2 independent experiments and actin expression (lower panels) is shown as a loading control.

Activation of NF-κB in certain cell types, including the ALCL cell line Karpas-299, is known to upregulate expression of p21^WAF1/CIP1^ [10]. To ascertain whether increased expression of p21^WAF1/CIP1^ downstream of D1 correlates with activation of NF-κB, the kinetics of IκBα phosphorylation and degradation were determined in 4-1BBL-stimulated Karpas-299 cell lines expressing the various chimeric receptors (Figure 3A). In all cells, except those transduced with p519, expression of phosphorylated IκBα peaked within 5–10 minutes of 4-1BBL incubation and IκBα was degraded and undetectable by 15 minutes showing that CD30 D1 and D2D3 independently activate the canonical NF-κB signaling pathway in ALCL cells. To characterise whether the alternative NF-κB pathway is engaged differentially by D1, D2 and/or D3, cells were stimulated with 4-1BBL or CD30L for 24 hours prior to detection of the p100/p52 NF-κB component (Figure 3B). Incubation of all transduced cell lines with CD30L resulted in degradation of p100 to the NF-κB subunit p52 as expected from previous reports [10], [32]. However after 4-1BBL incubation, p52 accumulation was only observed in cells expressing chimeric receptors incorporating D1 (i.e. cells transduced with pFL, pD1 or pD1D2). Indeed cells expressing a chimeric receptor encoding the entire cytoplasmic domain of human CD30 but lacking only D1 (pD2D3), failed to engage the alternative NF-κB pathway after incubation with 4-1BBL (Figure 3B), despite being fully competent for canonical NF-κB activation (Figure 3A). Michel cells transduced with pD1 similarly showed p52 accumulation following incubation with 4-1BBL (data not shown). These data demonstrate that activation of the canonical pathway can be instigated independently via either D1 or D2D3 whilst D1 is solely responsible for transmitting the signals required for activation of the alternative NF-κB pathway.

**Figure 4:**
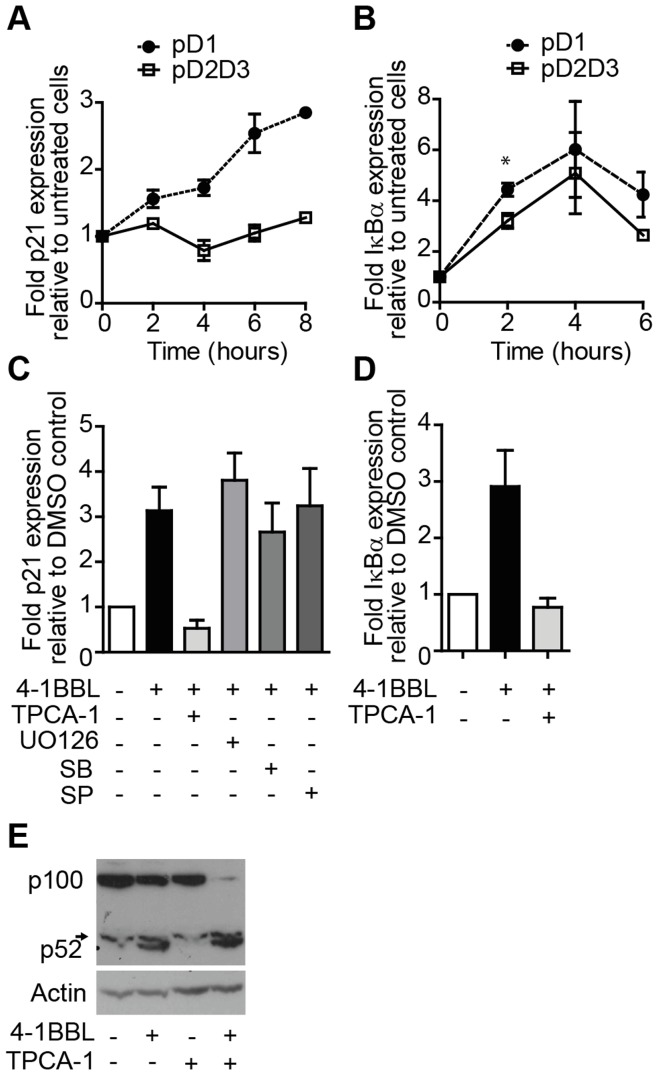
Differential regulation of NF-κB-dependent genes by CD30 D1 and D2D3. Karpas-299 cells stably transduced with pD1 (closed circles) or pD2D3 (open squares) were incubated with 4-1BBL, and p21^WAF1/CIP1^ (A) and IκBα (B) transcripts quantitated over time by qRT-PCR. Data show fold increase over untreated cells. (C and D) Karpas-299 pD1 transductants were incubated for 30 minutes with inhibitors of IKK-β (TPCA-1), ERK (UO126), p38 (SB203580; SB), JNK (SP600125; SP) or with an equal volume of DMSO (−), prior to addition of 4-1BBL where specified. After a further 7 hours p21^WAF1/CIP1^ (C) and IκBα (D) transcripts were quantitated by qRT-PCR. (E) In parallel whole cell lysates were assayed by immunoblot for p100/p52 and actin. Data are pooled from 2 (A and D) or 3 (B and C) experiments or are representative of 2 experiments (E). Graphs express the mean (+/−SEM)-fold increase in transcript expression over untreated (A and B) or DMSO-only treated (C and D) cells; *p<0.05 by two tailed Students t-test. The short arrow indicates a non-specific band above p52 (E).

Previously, upregulation of p21^WAF1/CIP1^ by the full length intracellular domain of CD30 was shown to be dependent on the canonical NF-κB pathway [10]. Figure 4A shows that the presence of the D1, but not D2D3, region was required for expression of the p21^WAF1/CIP1^ transcript, consistent with induction of the p21^WAF1/CIP1^ protein by this domain. In contrast, signals downstream of either D1 or D2D3 were sufficient to induce expression of IκBα, a known target of the canonical NF-κB pathway (Figure 4B). To investigate whether the canonical NF-κB pathway downstream of the D1 region is required for upregulation of p21^WAF1/CIP1^, we incubated cells with (TPCA-1), a selective inhibitor of IKKβ [33]. TPCA-1 completely ablated CD30 D1-induced upregulation of mRNA encoding p21^WAF1/CIP1^ and IκBα (Figure 4C and 4D respectively) confirming that transcription of both genes is dependent on the canonical NF-κB pathway. Of note, TPCA-1 did not prevent stimulation-induced degradation of p100 to p52 (Figure 4E), showing that activation of the alternative NF-κB pathway was not inhibited. As expected *de novo* synthesis of p100 was prevented by TPCA-1, confirming that p100 is itself a target of the canonical NF-κB pathway [34]. Finally, CD30 D1-induced p21^WAF1/CIP1^ expression was not suppressed by inhibitors of the JNK, ERK or p38 MAPK pathways (Figure 4C). Thus, while p21^WAF1/CIP1^ and IκBα transcription both require activation of the canonical NF-κB pathway which is triggered independently from both CD30 D1 and D2D3, only D1 is fully competent for p21^WAF1/CIP1^ transcription.

### The CD30 D1 Region Drives Increased Nuclear Accumulation of NF-κB p65 when Compared to D2D3

**Figure 5:**
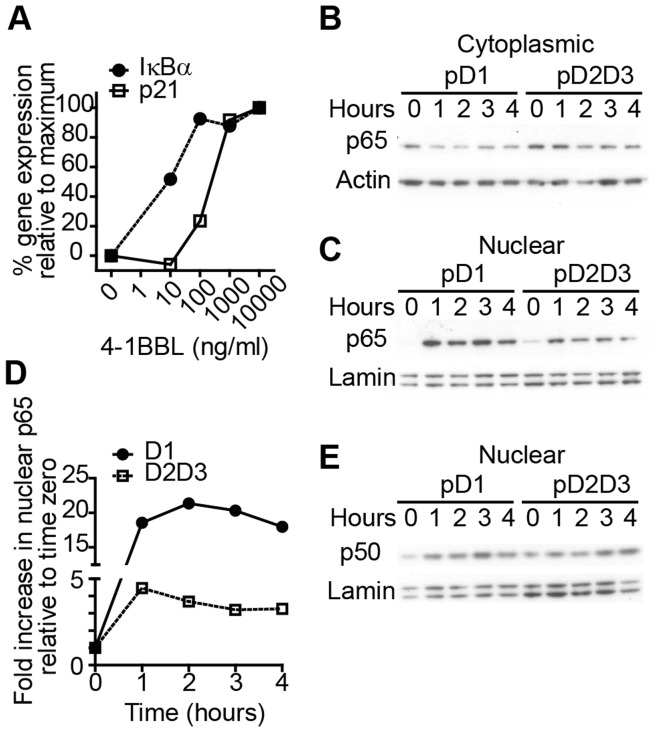
CD30 D1 triggers greater nuclear p65 accumulation than D2D3. (A) Karpas-299 cells stably transduced with pD1 were incubated with titrated doses of 4-1BBL for 5 hours and p21^WAF1/CIP1^ (open squares) and IκBα (closed circles) transcripts quantitated by qRT-PCR. Data represent the percentage fold-increase in expression relative to untreated (0%) and 10 µg/ml treated (100%) cells. (B, C, D and E) Karpas-299 cells stably transduced with pD1 or pD2D3 were incubated with 4-1BBL for the times indicated and cytoplasmic (B) or nuclear (C, D and E) fractions assayed by immunoblot for p65 (B, C and D), p50 (E) and actin and lamin as loading controls. D shows the fold-increase in nuclear p65 from pD1- (closed circles) and pD2D3- (open squares) expressing cells relative to time zero and normalised to lamin, calculated by densitometry and were obtained from a separate experiment to that shown in (C).

The *NFKB1A* gene which encodes IκBα incorporates 6 NF-κB-binding sites in its proximal promoter region, is exquisitely sensitive to the presence of nuclear NF-κB and is induced rapidly after receptor activation [35], [36]. In contrast, the *CDKN1A* gene encoding p21^WAF1/CIP1^ contains 2 κB-binding sites [31], [37] and is induced relatively late after receptor triggering. Given that transcription of late genes requires persistent nuclear NF-κB [35], [36], we hypothesised that greater accrual of nuclear p65 is required to drive transcription of p21^WAF1/CIP1^ and that this level is attained after signaling through D1 but not D2D3. To illustrate the differential sensitivity of IκBα and p21^WAF1/CIP1^ transcription following receptor signaling we compared expression of these two genes in cells stimulated with different concentrations of ligand. Figure 5A shows that when compared to IκBα transcription, initiation of p21^WAF1/CIP1^ transcription from D1 required at least a 10-fold higher concentration of the agonist ligand. This suggested that transcription of p21^WAF1/CIP1^ requires activation of a greater fraction of receptors when compared to transcription of IκBα, consistent with the notion that expression of p21^WAF1/CIP1^ requires increased nuclear concentrations of NF-κB. In Figure 5B we demonstrate that signals mediated through D1 caused cytoplasmic egress of p65 within 1 hour whereas D2D3-induced signaling was more protracted with decreased cytoplasmic p65 protein only detectable from 2–3 hours. In line with this kinetic profile, nuclear p65 accumulation was greater following D1 signaling compared with D2D3 (Figure 5C and D). The expression of nuclear p50 was similar from D1 and D2D3-expressors (Figure 5E). These data support our contention that NF-κB activation from D2D3 is sufficient only for IκBα transcription whereas increased nuclear p65 from D1 additionally drives expression of p21^WAF1/CIP1^.

## Discussion

We and others have reported previously that CD30 signaling in ALCL cells drives upregulation of the cyclin-dependent kinase inhibitor p21^WAF1/CIP1^ leading to growth arrest at the G_0_/G_1_ stage [8], [10], [12]. The data presented herein define a short region of the CD30 intracellular tail (D1; CD30_519–537_) that is responsible for mediating this effect. This same domain additionally activates the canonical NF-κB pathway and is necessary and sufficient for activation of the alternative NF-κB pathway. An additional membrane distal domain (D2D3; CD30_538–595_) independently activated only the canonical NF-κB pathway, but failed to induce upregulation of p21^WAF1/CIP1^. Our finding that D1 mediates substantial downstream signals stands in contrast with previous reports which together ascribe the majority of NF-κB activation from CD30 to CD30_538–595_ [14], [15]. However, two reports have described variable levels of NF-κB activation from domains similar to D1 (CD30_519–538_ [17] and CD30_524–531_ [16]) following transfection of HEK293 cells, albeit without addressing which NF-κB pathway is being activated or whether this region is required for induction of p21^WAF1/CIP1^. Additionally, ectopic expression of CD30 in HEK293 cells results in ligand-independent signaling [17] and thus may not be relevant to physiological activation of CD30. Our data extend previous findings by demonstrating that signals through CD30 D1 alone are sufficient to drive canonical and alternative NF-κB activation in cells that endogenously express CD30 and importantly that D1 signaling induces transcription of p21^WAF1/CIP1^ and impairs tumour cell division dependent on the canonical NF-κB pathway.

Our finding that independent functions can be ascribed to different regions of the CD30 cytoplasmic tail is reminiscent of aspects of CD40 signaling wherein alternative NF-κB activation requires a TRAF2/3 binding region but is independent of a TRAF6 binding domain [38]; conversely CD40-dependent polarization of macrophages towards an inflammatory phenotype requires TRAF6, but not TRAFs 2 or 3 [39]. For CD30, NF-κB signals emanating from D2D3 are likely mediated via TRAFs 2 and 5 [15]. However, in light of reports that TRAF proteins bind only to the D2D3 domain, it is unclear how D1 is able to engage downstream signaling pathways [13]–[16]. Duckett *et. al.* (1997) previously proposed that CD30_410–531_, which incorporates all but 7 residues of D1, may represent a death domain (DD)-like region analogous to those found in the cytoplasmic domains of other members of the TNFRSF e.g. TNFR1, Fas and DR3 [16]. Recruitment of TRADD, RIP1 and TRAF2 to the DD of TNFR1 or DR3 drives NF-κB activation with TRADD acting as a bridge for TRAF2 binding [40]–[43]. Whether D1 binds to DD-containing proteins or other protein(s) not currently associated with classical TNFRSF signaling pathways remains to be determined. The requirement for TRAF family members further downstream of D1 is also controversial; one report documents complete abrogation of NF-κB signaling from CD30_500–538_ by dominant negative versions of TRAFs 2 or 5 [17] whereas others have shown no deleterious effects of dominant negative TRAFs 1, 2 or 3 on NF-κB activation arising from CD30_410–531_ (incorporating D1 but not D2D3) signaling in HEK293 cells [16]. In our hands, Karpas-299 cells stably transduced with dominant negative TRAF2 continue to undergo growth arrest and activate the alternative NF-κB pathway after CD30L incubation (data not shown) lending support for a TRAF-independent mode of signal transduction from D1 in ALCL cells.

An obvious question arising from our results concerns how both D1 and D2D3 domains can induce canonical NF-κB activation and transcription from *NFKB1A*, yet only D1 is fully competent for driving transcription of the NF-κB-dependent *CDKN1A* gene. The *NFKB1A* gene is an ‘early gene’ reflecting the rapid kinetics of its transcription by NF-κB, and as such is upregulated rapidly in response to low levels of receptor agonist in a digital (on/off) manner. In contrast genes exhibiting a slower kinetic of expression such as p21^WAF1/CIP1^, respond with a more analogue profile, being more sensitive to both initial dose and stimulus persistence [36]. Our data are entirely in keeping with these findings and indicate that the lower initial nuclear concentration and short-lived nuclear p65 seen following D2D3 signaling is probably sufficient only for IκBα transcription whereas D1-induced p65 accumulation is of sufficient magnitude to additionally allow for p21^WAF1/CIP1^ upregulation and growth arrest. The question that then arises is why should p65 nuclear import be greater after D1 engagement compared with D2D3? CD30 interacts with the aryl hydrocarbon nuclear translocator (ARNT) via the D2D3 region which is thought to be part of a negative feedback pathway that interferes with recruitment of TRAFs to the D2D3 region, thus suppressing NF-κB activation downstream of CD30 [44]. Because D1 does not interact directly with either TRAFs or ARNT, the signals that activate NF-κB emanating from D1 are less likely to be subject to negative regulation by ARNT, providing a potential explanation for the higher levels of NF-κB activation downstream of D1.

In addition it is plausible that concurrent activation of the alternative NF-κB pathway by D1 may augment the magnitude of canonical NF-κB signaling. Indeed several aspects of the alternative NF-κB pathway can act in this manner. For example, loss of TRAF3, which occurs within 30 minutes of CD40 or BAFFR stimulation and which is necessary for NIK accumulation [20], [21], additionally permits increased p65/p50 nuclear import and transcription of NF-κB target genes after TNF-α stimulation [23]. In addition, knock-down of NIK abrogates not only CD27-driven p100 processing to p52 but also IκBα degradation and nuclear p65 accumulation [24] while NIK stabilisation results in loss of IκBα [22] indicating a positive role for NIK in canonical NF-κB activation. Furthermore, as well as targeting p100 for proteolysis, activated IKKα drives phosphorylation of both IκBα and IKKβ, the latter serving to further enhance IκBα degradation [45], [46]. Finally, dimers of p100 have been shown to retain p65/p50 complexes in the cytosol of resting cells thereby inhibiting their DNA binding activity. On activation of the alternative NF-κB pathway p100 is processed to p52 thereby releasing the bound p65/p50 transcription factor and driving canonical NF-κB activation [47]. A mathematical model which takes this additional role of p100 into account predicts that activation of the canonical pathway alone will drive a high magnitude but transient RelA/p50 nuclear signal which returns to base level by 2 hours, whereas a receptor which triggers only the alternative pathway induces a low but gradual increase in canonical NF-κB activity over time [47]. This finding fits with our results in which D2D3 activates transcription of the sensitive *NFKB1A* gene only, whereas D1 promotes enhanced nuclear accumulation of p65 favouring additional p21^WAF1/CIP1^ transcription. Although activation of the alternative NF-κB pathway is considered prolonged compared with the canonical pathway, p100 degradation can be detected as early as 20 minutes after CD27 stimulation confirming that loss of p100 early during alternative NF-κB activation might influence the magnitude of simultaneous canonical NF-κB activation [24], [47].

Our data also show that blockade of IKKβ inhibits the transcription of p21^WAF1/CIP1^, but not the processing of p100 downstream of D1, confirming the canonical NF-κB dependency of p21^WAF1/CIP1^ transcription. A previous report has similarly shown RelA (p65), but not p100, to be an important driver of p21^WAF1/CIP1^ transcription downstream of full length CD30 in ALCL cells [10]. Interestingly, depletion of p100 slightly increased p21^WAF1/CIP1^ expression in that report [10] thereby ruling out an essential role for p52 in driving p21^WAF1/CIP1^ transcription from D1 and instead providing support for an IκB-type function of p100 as described above. Furthermore, p100 and p65 associate in resting Karpas-299 cells [32], further suggestive of a potential inhibitory role of p100 on canonical NF-κB activation from CD30 in these cells.

Finally, our finding that distinct functions of CD30 are attributed to discrete cytoplasmic regions suggests a mechanism to explain how CD30 engagement leads to different cellular outcomes. Specifically, we propose that alternative utilization of these cytoplasmic motifs in different cell types, or as a result of CD30 engagement by different agonists, is responsible for the multiple and often opposing roles of CD30 [3]–[9].

## Supporting Information

Figure S1 **Expression of 4-1BB on the surface of transduced Michel cells.** Michel ALCL cells were stably retrovirally transduced with the plasmids indicated and surface expression of 4-1BB confirmed by flow cytometry. Plots show staining with isotype control (filled histograms) or with anti-4-1BB (open histograms) antibody.
(TIF)

Figure S2 **Signaling through CD30 induces minimal apoptosis.** Karpas-299 cells retrovirally transduced with the receptors indicated were incubated alone or with 4-1BBL for 24 hours prior to analysis of DNA content by propidium iodide staining. Numbers indicate the percentage of cells undergoing apoptosis (left) or in S-phase (right).
(TIF)
