# Supplementary figures and images for: Distinct Motifs in the Intracellular Domain of Human CD30 Differentially Activate Canonical and Alternative Transcription Factor NF-κB Signaling
Source: PLoS One. 2012 Sep 18;7(9):e45244. doi: 10.1371/journal.pone.0045244 (PMC3445475; doi:10.1371/journal.pone.0045244)

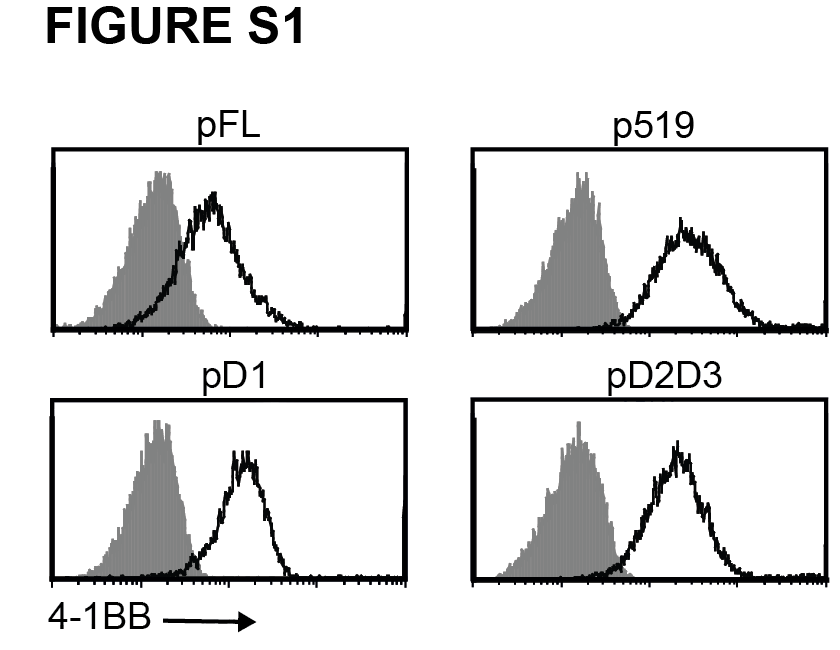

Supplement: Figure S1 — Expression of 4-1BB on the surface of transduced Michel cells. Michel ALCL cells were stably retrovirally transduced with the plasmids indicated and surface expression of 4-1BB confirmed by flow cytometry. Plots show staining with isotype control (filled histograms) or with anti-4-1BB (open histograms) antibody. (TIF) [file pone.0045244.s001.tif]

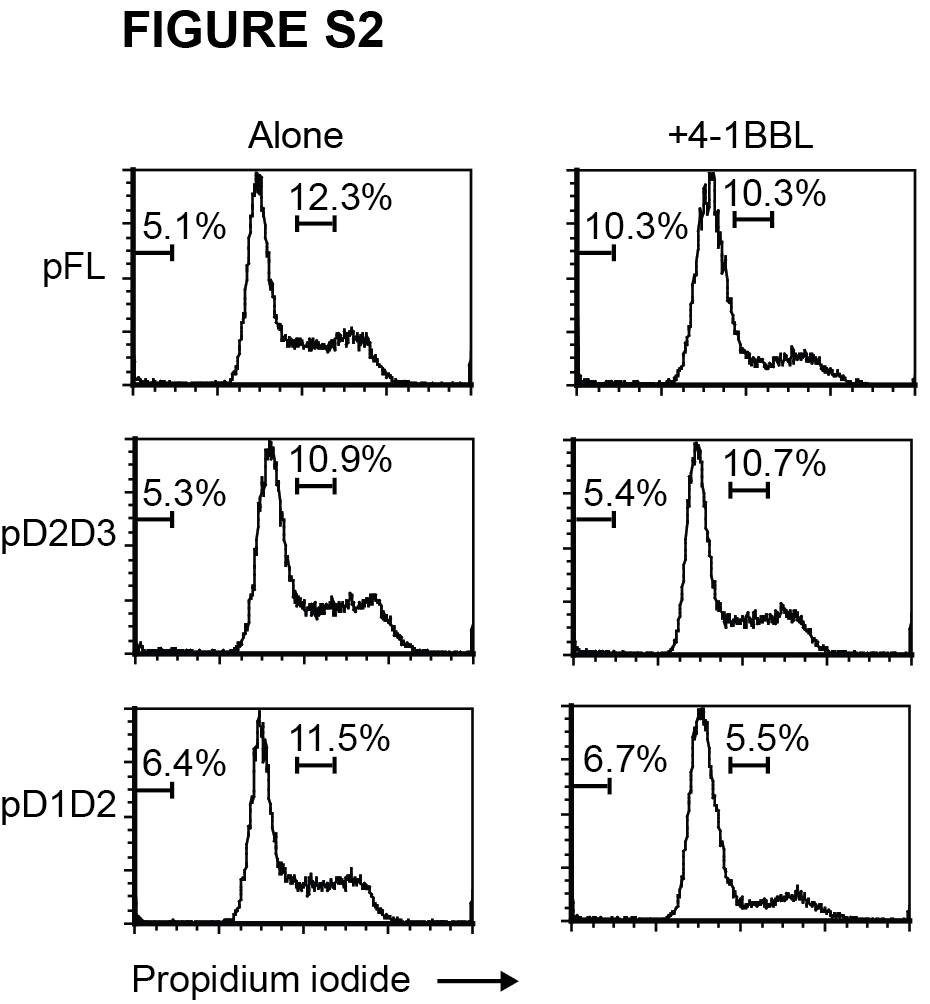

Supplement: Figure S2 — Signaling through CD30 induces minimal apoptosis. Karpas-299 cells retrovirally transduced with the receptors indicated were incubated alone or with 4-1BBL for 24 hours prior to analysis of DNA content by propidium iodide staining. Numbers indicate the percentage of cells undergoing apoptosis (left) or in S-phase (right). (TIF) [file pone.0045244.s002.tif]
